# Health-promoting effects of *Clostridium butyricum* GKB7 on the gastrointestinal tract in murine models

**DOI:** 10.1016/j.bbrep.2025.102145

**Published:** 2025-07-11

**Authors:** You-Shan Tsai, Chia-Chi Chen, Li-Ya Lee, Shih-Wei Lin, Yen-Lien Chen, Chin-Chu Chen

**Affiliations:** a Biotech Research Institute, Grape King Bio Ltd., Taoyuan City, Taiwan; b Animal Technology Research Center, Agricultural Technology Research Institute, Hsinchu City, Taiwan; c Institute of Food Science and Technology, National Taiwan University, Taipei City, Taiwan

**Keywords:** *Clostridium butyricum*, Constipation, Inflammatory bowel disease, Peptic ulcer disease, Probiotics

## Abstract

*Clostridium butyricum* is an anaerobic bacterium known for its ability to produce butyrate and modulate gut microbiota. This study aimed to evaluate the protective and regulatory effects of a novel strain, *Clostridium butyricum* GKB7, isolated from a healthy Taiwanese individual, on gastrointestinal tract. Three rodent disease models were established: picrylsulfonic acid-induced colitis in rats, loperamide-induced constipation in rats, and aspirin-induced gastric ulcers in mice. Strain GKB7 was administered orally at doses equivalent to human intake with 100 mg/60kg/day. It was found that strain GKB7 significantly normalized colon length and weight, reduced intestinal injury, and partially protected the enterochromaffin cell in colitis model. Besides, strain GKB7 improved stool water content over time and significantly enhanced gastrointestinal motility in constipation model. Furthermore, strain GKB7 mitigated gastric ulcer severity and tissue damage, achieving a 25.2 % curative ratio in gastric ulcer model, with observed reductions in ulcer depth, area, and inflammation. *C*. *butyricum* GKB7 exhibits probiotic potential by improving colonic integrity, promoting bowel movement, and protecting against gastric injury. These results support its potential as a supplement for gastrointestinal disorders including inflammatory bowel disease, constipation, or peptic ulcers.

## Highlights

• *C. butyricum* GKB7 reduces colonic inflammation and supports mucosal integrity.
• Administration of strain GKB7 significantly enhances gastrointestinal motility.
• Strain GKB7 reduces NSAID-induced gastric damage, potentially through mucosal protection.
• The dosage of GKB7 falls within the human intake range, highlighting its translational potential.

## Graphical abstract

**Figure:**
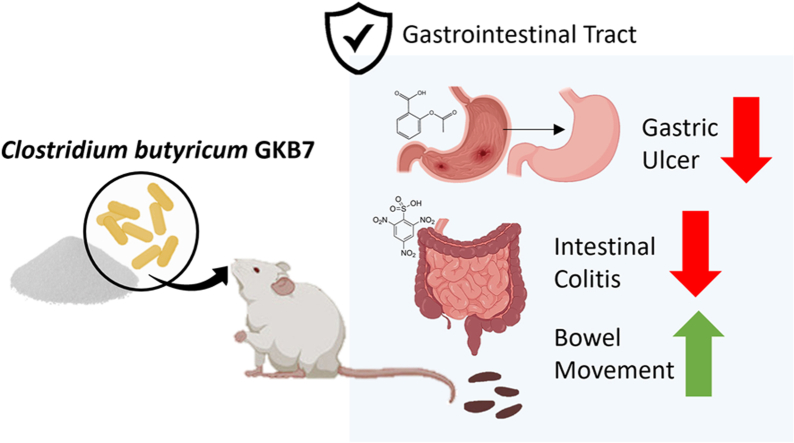

## 1 Introduction

Gastrointestinal disorders such as peptic ulcers, inflammatory bowel disease, and constipation are prevalent conditions that significantly impact quality of life [1,2]. These conditions are often characterized by disruptions to the gut epithelial barrier, microbial dysbiosis, and chronic inflammation [3]. Despite advances in conventional therapies, many treatments are associated with adverse effects or limited efficacy in restoring gut homeostasis [4]. Probiotics, defined as live microorganisms that confer health benefits when administered in adequate amounts, have emerged as promising alternatives due to their potential in modulating gut microbiota, enhancing mucosal immunity, and producing beneficial metabolites such as short-chain fatty acids (SCFAs) [[5], [6], [7]].

*Clostridium butyricum* is a strictly anaerobic, Gram-positive, spore-forming bacterium found in the normal gut flora of humans and animals [[8], [9], [10]]. It was recognized for its ability to generate butyrate – an essential SCFA for colonic epithelial cells with anti-inflammatory properties [11,12]. Recent studies have demonstrated its potential in alleviating metabolic syndrome, osteoarthritis, and other inflammation-related gut-organ axis [[13], [14], [15]]. Although the beneficial effects of *C*. *butyricum* have been reported, strain-specific differences may result in pathogenic outcomes [16,17]. After all, closely related species such as *C*. *perfringens* and *C*. *botulinum* can be known causes of foodborne illnesses, while *C*. *difficile* is a major pathogen responsible for colonic infections [18,19]. Given this background, a novel strain *C*. *butyricum* GKB7, isolated from a healthy Taiwanese and selected for its high butyrate-producing capacity, warrants further investigation for its effects on the gastrointestinal tract [20].

In this study, we investigated strain GKB7 by three rodent models mimicking human gastrointestinal disorders: colitis, constipation, and gastric ulcer. Through a combination of physiological and histological assessments, we aimed to elucidate the protective and regulatory effects of strain GKB7 on gut health.

## 2 Material and methods

### 2.1 Bacterial sources

*Clostridium butyricum* GKB7 (GKB7) was isolated from the gut microbiota of a healthy Taiwanese individual and provided by Grape King Bio Ltd. (Taoyuan, Taiwan). The bacterial cell count was approximately 1.0 × 10^10^ CFU/g, as determined using Clostridia Count Agar (CCA; Nissui, Tokyo, Japan). The dosage used in the following studies was equivalent to 100 mg/day for a 60-kg human, corresponding to 20.5 mg/kg/day for mice and 10.33 mg/kg/day for rats based on body surface area conversion.

### 2.2 Animal handling

Sprague-Dawley rats and ICR mice were purchased from BioLASCO Taiwan Co., Ltd. (Taipei, Taiwan) and maintained under controlled conditions: 18–26 °C, 30–70 % humidity, and a 12-h light/dark cycle. Food and water were provided *ad libitum*. Animals were housed and acclimated for at least one week prior to the commencement of the experiments. All experimental protocols were approved by the Institutional Animal Care and Use Committee (IACUC) of the Agricultural Technology Research Institute (ATRI, Hsinchu, Taiwan), under the following approval numbers: No. 112058 for the TNBS-induced colitis model, No. 112019 for the loperamide-induced constipation model, and No. 110075 for the aspirin-induced gastric injury model.

### 2.3 TNBS-induced colitis model

**Fig. 1:**
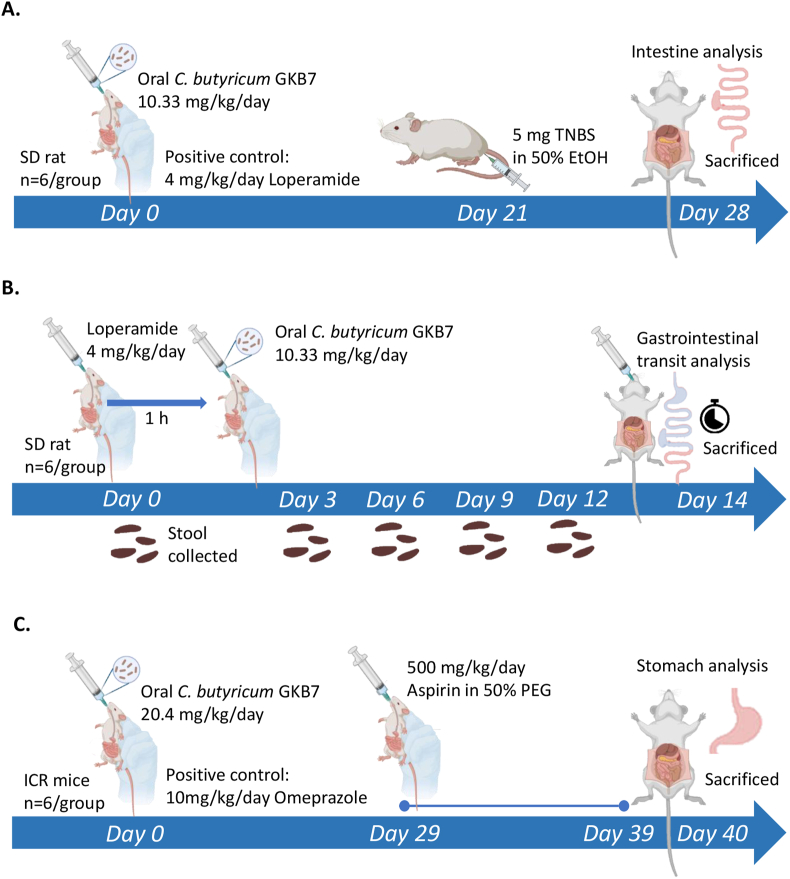
**Study designs:** (A) TNBS-induced colitis model; (B) Loperamide-induced constipation model; (C) Aspirin-induced gastric injury model.

Picrylsulfonic acid solution (2,4,6-trinitrobenzene sulfonic acid, TNBS; Cat. No. SI–P2297) was purchased from Sigma-Aldrich Co. (MA, USA) and used to induce colitis at a dose of 5 mg/mL in 50 % ethanol [21]. Eight-week-old male SD rats, approximately 250 g each, were randomly divided into four groups (n = 6 per group): (1) normal control, (2) TNBS-induced colitis (negative control), (3) TNBS-induced colitis treated with loperamide (positive control, 4 mg/kg/day), and (4) TNBS-induced colitis treated with strain GKB7. From day 0 to day 27, rats in the normal and TNBS groups received drinking water via oral gavage, while the treatment groups received either loperamide or GKB7. On day 21, all groups except the normal control received intrarectal administration of TNBS. The normal group received an equal volume of saline instead. On day 28, all rats were sacrificed, and colons were collected for further analysis (Fig. 1A).

### 2.4 Loperamide-induced constipate model

Loperamide hydrochloride (Cat. No. L4762, Sigma-Aldrich, MA, USA) was administrated to inhibit intestinal motility at a dose of 4 mg/kg/day [22]. Eight-week-old male SD rats were randomly dived into three groups (n = 6 per group): (1) normal group, (2) loperamide group (negative control), and (3) loperamide-induced constipation group treated with strain GKB7. Expect for the normal group, all rats received loperamide via oral gavage from day 0 to day 13. Probiotics GKB7 was administered daily from day 0 to day 13. Stool samples were collected on days 0, 3, 6, 9, and 12. On day 14, a gastrointestinal transit assay was conducted, then all the rats were sacrificed (Fig. 1B).

### 2.5 Aspirin-induced gastric ulcer model

Acetylsalicylic acid (aka Aspirin, Cat. No. A5376) was purchased from Sigma-Aldrich Co. (MA, USA) and used to induce gastric ulcer [23]. Eight-week-old male ICR mice, approximately 25 g each, were randomly assigned to four groups (n = 6 per group): (1) normal control, (2) Aspirin-induced gastric injury group (negative control), (3) Aspirin-induced gastric injury treated with Omeprazole (positive control, 10 mg/kg/day) [24], and (4) Aspirin-induced gastric injury treated with strain GKB7. From day 0 to day 39, mice in the normal and Aspirin groups received drinking water via oral gavage, while the treatment groups received either Omeprazole or GKB7. From day 29 to day 39, all groups except the normal control were administrated 500 mg/kg/day Aspirin via oral gavage to induce gastric injury (Fig. 1C). On day 40, all mice were sacrificed and stomach tissues were collected for further analysis.

### 2.6 Stool sampling

Stool samples from the loperamide-induced constipation model were collected and weighted to obtain the wet weight. The samples were then dried at 60 °C for 24 h and reweighted to determine the dry weight. Stool water content was calculated using the following formula:

$$Stool\;water\;content\;\left(\%\right)=\left(\frac{wet\;weight-dry\;weight}{wet\;weight}\right)\times 100$$

### 2.7 Gastrointestinal transit (GIT) assay

A total of 1.5 g Coomassie brilliant blue R-250 (Cat. No. B0149, Sigma-Aldrich Co., MA, USA) and 0.25 g carboxymethylcellulose (CMC; Cat. No. 21902, Sigma-Aldrich Co., MA, USA) were dissolved in 50 mL of drinking water to prepare a marker blue CMC solution for the gastrointestinal transit (GIT) assay [25]. One hour before sacrifice, rats were orally gavaged with 1 mL of the marker solution. After sacrifice with an intramuscular injection of Zoletil 50 (50 mg/mL; Cat. No. 5TK3, Virbac Laboratories, Carros, France), the entire intestine was excised, and the distance traveled by the blue dye was measured. The GIT ratio was calculated using the formula:

$$Gastrointestinal\;transit\;ratio\;\left(\%\right)=\left(\frac{distance\;traveled\;by\;blue\;CMC}{total\;length\;of\;intestine}\right)\times 100$$

### 2.8 Gastrointestinal injury area, gastric index, and curative ratio

Colonic or gastric injury areas were quantified using ImageJ software. To determine the gastric ulcer index (UI), lesions were categorized into three levels based on injury area <1 mm^2^ for level I, 1–3 mm^2^ for level II, and >3 mm^2^ for level III. The UI was calculated using the following formula:

$$Ulcer\;Index\;\left(UI\right)=\frac{\left(1\times number\;of\;level\;I\right)+\left(2\times number\;of\;level\;II\right)+\left(3\times number\;of\;level\;III\right)}{total\;number\;of\;mice\;in\;the\;group}$$

The curative ratio was determined by comparing the ulcer index with the following formula:

$$C\text{urative}\;\text{ratio}\;\left(\%\right)=100-\left(\frac{UI\;from\;treatment\;group}{UI\;from\;negative\;control}\times 100\right)$$

### 2.9 Histopathological examination

Colonic or gastric tissues were fixed in 10 % formalin overnight, dehydrated through graded ethanol and xylene, embedded in paraffin, and sectioned at 4 μm thickness. Hematoxylin and eosin (H&E) staining was performed for histological examination of gastric tissues. Based on previous studies, pathological scoring was assessed under 100 × magnification on a scale from 0 (normal) to 4 (severe), according to ulcer depth, ulcer area, degree of inflammation, and tissue regeneration [26].

### 2.10 Immunohistochemistry staining

To quantify enterochromaffin (EC) cells in colonic tissues, immunohistochemical (IHC) staining were performed [27]. Paraffin-embedded sections were deparaffinization and rehydration, followed by antigen retrieval, blocking of endogenous peroxidase activity, and nonspecific binding. Sections were then incubated overnight at 4 °C with an anti-Chromogramin A antibody (CgA, 1:200 dilution; Cat No. A9576, ABclonal Technology, MA, USA), followed by the ImmPRESS® HRP anti-rabbit IgG polymer detection kit (Cat. No. MP-7401, Vector Labs, CA, USA), and visualized using DAB chromogen. Slides were counterstained with hematoxylin and observed under 200 × magnification using light microscope (BX51, Olympus, Tokyo, Japan) to count EC cells (CgA^+^).

### 2.11 Statistics

Data analysis and visualization were conducted using GraphPad Prism version 10 (GraphPad Software, CA, USA). Results are presented as the mean ± standard deviation (SD). Statistical significance was determined using one-way ANOVA, with comparisons made against the negative control group. Significance levels were indicated as ∗*p* < 0.05, ∗∗*p* < 0.01, and ∗∗∗*p* < 0.001.

## 3 Results

### 3.1 Protection effects of Clostridium butyricum GKB7 in colitis

**Fig. 2:**
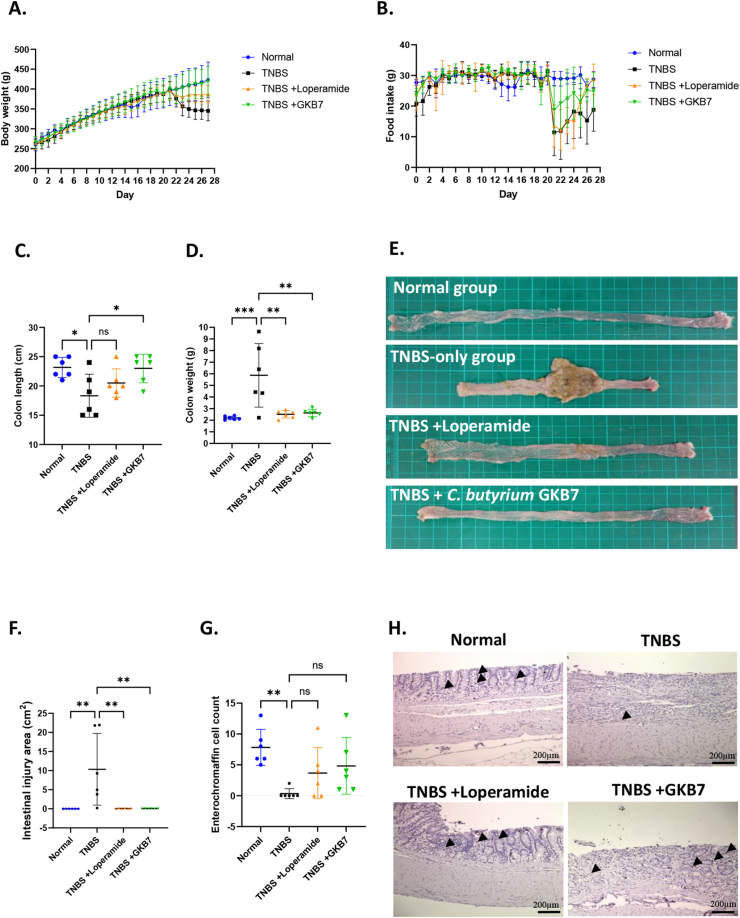
**Protective effects of *Clostridium butyricum* GKB7 on intestinal integrity in a TNBS-induced rat model.** (A) Daily body weight changes; (B) Daily food intake; (C) Colon weight; (D) Colon length; (E) Appearance of the intestine; (F) Intestinal injury area; (G) Enterochromaffin cell count; (H) IHC staining of colon tissue, arrows indicate enterochromaffin cells stained brown. Data are presented as mean ± SD (n = 6). Statistical significance was determined by one-way ANOVA, with comparisons made against the TNBS group. Differences were considered significant at *p* < 0.05.

Colitis was induced in rats using TNBS. After induction, rats received TNBS exhibited body weight loss and less food intake. In contrast, administered with strain GKB7 demonstrated weight gain comparable to the normal group, despite a decline in food intake starting from day 21 (Fig. 2A). Notably, the GKB7 group showed a more favorable recovery in food consumption compared to the TNBS-only group (Fig. 2B).

A significant decrease in colon length and an increase in colon weight were observed in the TNBS-only group compared to the normal group. While loperamide administration partially reversed the increase in colon weight (Fig. 2D), it did not significantly restore colon length compared to the TNBS-only group (Fig. 2C). In contrast, treatment with strain GKB7 significantly reversed both the colon length and colon weight (Fig. 2E).

Furthermore, the TNBS-only group exhibited significantly larger areas of colonic injury. In contrast, administration of loperamide or strain GKB7 conferred protective effects, resulting in reduced injury areas (Fig. 2F). EC cells, which are specialized neuroendocrine cells in the gastrointestinal tract, were markedly decreased following TNBS induction (Fig. 2G). Although no statistically significant differences in EC cell counts were observed in neither loperamide treatment nor GKB7 group, an increasing trend of cell number was noted in the GKB7-treated group (Fig. 2H).

### 3.2 Enhancement of bowel functions of Clostridium butyricum GKB7

**Fig. 3:**
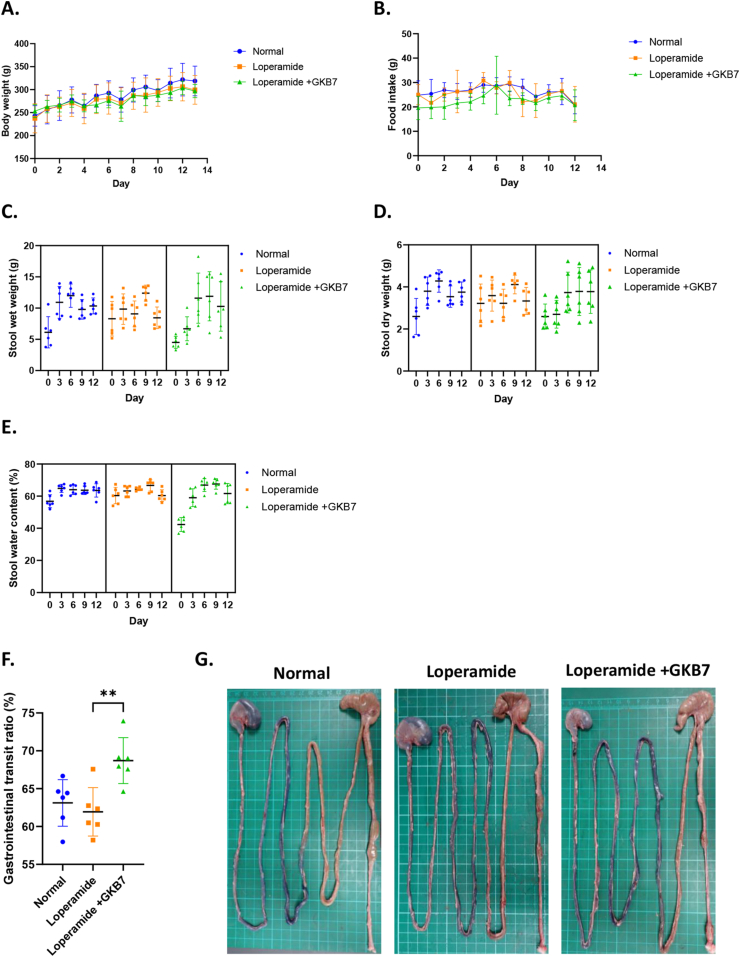
**Effects of *C. butryrium* GKB7 on growth and intestinal function in a Loperamide-induced constipation rat model.** (A) Daily body weight changes; (B) Daily food intake; (C) Stool wet weight; (D) Stool dry weight; (E) Stool water content; (F) Gastrointestinal transit ratio; (G) Intestine appearance following Coomassie blue. Data are presented as mean ± SD (n = 6). Statistical significance was determined by one-way ANOVA, with comparisons made against the Loperamide group. Differences were considered significant at *p* < 0.05.

Constipation was induced in rats by administering loperamide to reduce bowel motility. No significant differences in body weight (Fig. 3A) or food intake (Fig. 3B) were observed among the groups throughout the experiment. Stool samples were collected on days 0, 3, 6, 9, and 12. The wet and dry weights of stool samples were presented in Fig. 3C and D, respectively. On day 12, no significant differences in stool wet or dry weights were found between the loperamide-only and GKB7 groups. Notably, the initial stool wet weight in the loperamide-only group was relatively higher than that in the GKB7 group. Stool water content showed no statistical differences among the three groups on day 12 (Fig. 3E); however, the GKB7 group initially exhibited significantly lower stool water content compared to the loperamide-only group or normal group. When comparing stool water content between the first and the second week, an increasing trend was observed in the GKB7 group (Table S1).

Blue CMC was applied as a marker to assess intestinal motility (Fig. 3G). There was no significant difference in the GIT ratio between the normal and loperamide-only groups. However, administration of strain GKB7 in the loperamide-induced group significantly enhanced gastrointestinal motility (Fig. 3F).

### 3.3 Protection effects of Clostridium butyricum GKB7 in stomach

**Fig. 4:**
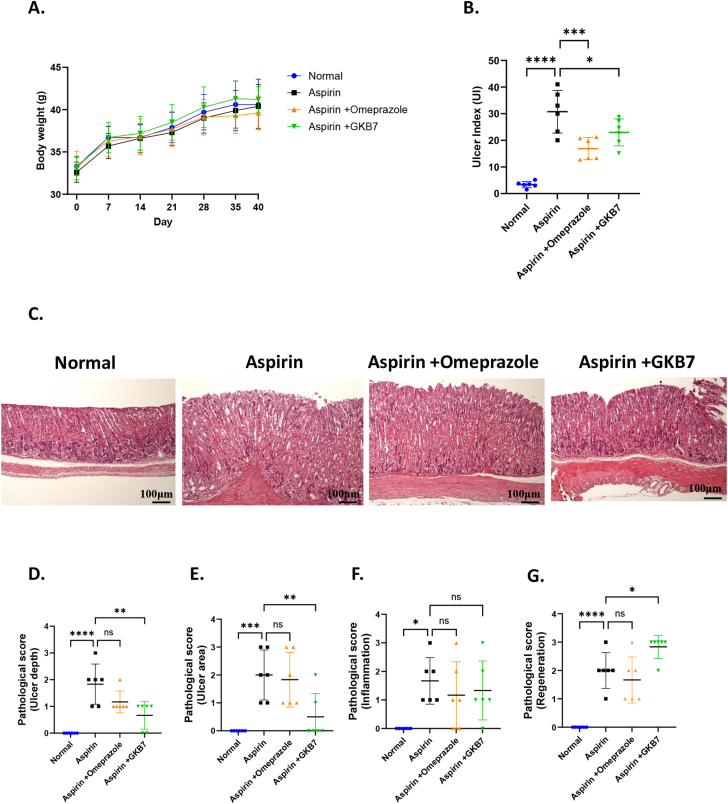
**Protective effects of *Clostridium butyricum* GKB7 on the stomach in an Aspirin-induced gastric injury mice model.** (A) Body weight changes; (B) Ulcer index; (C) Representative images of stomach tissue; (D–G) Pathological scores of stomach tissue, including (D) lesion depth, (E) ulcer area, (F) inflammation, and (G) tissue regeneration. Data are presented as mean ± SD (n = 6). Statistical significance was determined by one-way ANOVA, with comparisons made against the Aspirin group. Differences were considered significant at *p* < 0.05.

Gastric ulcers were induced in mice using aspirin. No significant differences in body weight were observed among the groups (Fig. 4A). After 10 days of aspirin administration, both the ulcerated area and the number of ulcers in the stomach significantly increased (Table S2). The ulcer index was markedly elevated in the aspirin-only group, whereas treatments with omeprazole or strain GKB7 significantly reduced the ulcer severity (Fig. 4B). The curative ratios for omeprazole and strain GKB7 were 44.99 % and 25.20 %, respectively.

Histopathological examination of stomach tissues revealed mucosal epithelial cell damage and necrosis in the aspirin-only group (Fig. 4C). In contrast, the integrity of the stomach tissue was better preserved in the strain GKB7 group. Specifically, strain GKB7 demonstrated protective effects by reducing ulcer depth (Fig. 4D), ulcer area (Fig. 4E), and showing mild anti-inflammatory activity (Fig. 4F). However, noticeable regeneration of mucosal epithelial cells was not observed in the GKB7 group (Fig. 4G).

## 4 Discussion

This study demonstrated the multifunctional gastrointestinal benefits of *C*. *butyricum* GKB7. TNBS was commonly used to demonstrate the disease symptoms of colitis, the clinical signs usually included the body weight loss, soft stools, or diarrhea [28]. The mechanism of TNBS involves the induction of an IL-12-mediated Th1 response and the activation of inflammatory signaling pathways, leading to colonic tissue injury [29]. Both the histological and immunological features closely resemble those observed in human inflammatory bowel disease. In the TNBS-induced colitis model, administration of strain GKB7 significantly alleviated key pathological features associated with colonic inflammation (Fig. 2C and D). Improvements in colonic appearance and a trend toward increased enterochromaffin cell number suggested that GKB7 may exert both structural and functional protective effects on the inflamed colon (Fig. 2E and H). These findings are consistent with previous reports on other *C. butyricum* strains, which have demonstrated anti-inflammatory effects in colitis models through modulation of cytokine profiles and maintenance of epithelial barrier function [30,31].

Notably, although the increase in EC cells did not reach statistical significance, the upward trend implies that strain GKB7 may contribute to the normalization of intestinal permeability and gastrointestinal motility (Fig. 2G). EC cells are responsible for 5-hydroxytryptamine (5-HT) synthesis, storage and release of the gut [32]. Since the 5-HT, also known as serotonin, involved with intestinal enteric nervous system and muscular movements, the depletion of EC cells is commonly observed in inflammatory bowel diseases [33,34]. This may be attributed to 5-HT released from EC cells can stimulate goblet cells to secrete mucus, regulate enteric neurons involved in pain or peristalsis, and modulate immune cell responses to promote anti-inflammatory effects. [35]. Therefore, preservation of EC cells may represent a benefit of strain GKB7 in intestinal integrity. However, larger-scale studies are needed to confirm these preliminary findings and clarify the functional implications of EC cell modulation by GKB7.

Loperamide, an opioid receptor agonist, is commonly used to alleviate diarrhea symptoms in patients with inflammatory bowel disease [36]. However, constipation is one of its known side effects, primarily due to the activation of μ-opioid receptors in the myenteric plexus [37]. This interaction decreases the tone of both longitudinal and circular smooth muscles in the intestine, thereby slowing gastrointestinal motility. In the loperamide-induced constipation model, strain GKB7 significantly enhanced gastrointestinal transit, despite the absence of statistically significant differences between the normal group and the loperamide-only group (Fig. 3F). It is suggested that strain GKB7 might also enhance functional motility in healthy subjects (Fig. 3H). Since the stool water content was significantly lower in the GKB7 group at the first week (Table S1), an increased water content resulting stool bulk-forming effects was eliminated at the finial observation when compared to the loperamide-only group (Fig. 3C and D). The observed progressive increase in stool water content over time in strain GKB7 group further supports a time-dependent promoting pattern (Fig. 3E). This pattern mirrors observations in the probiotic study by Inatomi and Honma [22], supporting the potential of GKB7 to attenuate loperamide-induced constipation over time, primarily by promoting intestinal motility and partially restoring stool hydration.

Butyrate, a key metabolite produced by *Clostridium butyricum*, has been shown to stimulate colonic motility by activating enteric neurons via monocarboxylate transporter 2 (MCT2), thereby playing a beneficial role in gastrointestinal transit [38]. In contrast, other SCFAs such as acetate and propionate, primarily generated by different microbial species, might exert inhibitory effects on colonic motility [39]. Therefore, not only the types and concentrations of SCFAs but also the composition of the gut microbiota are critical determinants of intestinal motility. It is possible that the motility-enhancing effects observed with strain GKB7 were mediated through similar mechanisms, potentially involving the restoration of microbial balance and the modulation of SCFAs profiles, either directly or indirectly via interaction with EC cells or enteric neurons.

Non-steroidal anti-inflammatory drugs (NSAIDs) such as aspirin and ibuprofen are widely used to relieve pain associated with fever, menstruation, and dental procedures [40]. Aspirin inhibits cyclooxygenase (COX) enzymes, where COX-2 is involved in regulating inflammatory responses, while COX-1 plays a role in maintaining the normal function of the gastrointestinal system. Since aspirin inhibits both COX-2 and COX-1, gastric injury is one of its common side effects [41]. In the aspirin-induced gastric ulcer model, strain GKB7 significantly reduced gastric ulcer severity, with a curative ratio of 25.2 % (Fig. 4B). Histological analysis further revealed decreased ulcer depth, area, and mildly anti-inflammation, suggesting a mucosal protective role (Fig. 4D–F). These protective effects may be attributed to anti-inflammatory activity and enhancement of mucosal defenses, consistent with previous findings where *C. butyricum* suppressed inflammatory cytokines and oxidative stress under NSAID intervention [42]. Moreover, the stomach-protective effects observed with GKB7 in this model align with our earlier findings in an alcohol-induced gastric injury model, suggesting that strain GKB7 may offer broad protective potential against various gastric stressors [43].

Together, these results highlight the multifaceted gastrointestinal benefits of *Clostridium butyricum* GKB7, including anti-inflammatory effects in the colon, pro-motility activity in the small and large intestines, and potential mucosal protection in the stomach. However, this study was limited by the absence of analyses on gut microbiota composition, cytokine expression, and gene or protein-level responses to GKB7 intervention. Further investigations are needed to elucidate the underlying mechanisms, particularly those related to epithelial barrier integrity. Importantly, the dosage applied in this study corresponds to a feasible daily intake for humans, reinforcing its translational potential. Future studies are warranted to confirm these findings in human clinical trials.

## 5 Conclusion

In this study, we demonstrated that *Clostridium butyricum* GKB7 confers protective and regulatory effects across multiple gastrointestinal disorders in rodent models, including TNBS-induced colitis, loperamide-induced constipation, and aspirin-induced gastric ulcer. Strain GKB7 effectively mitigated colonic injury, improved intestinal motility, and reduced gastric damage. These findings support the strain GKB7 in promoting gut health through anti-inflammatory, pro-motility, and mucosal-protective mechanisms. Future research should focus on its molecular mechanisms, long-term safety, and efficacy in human clinical settings to further validate its potential for clinical application.

## CRediT authorship contribution statement

**You-Shan Tsai:** Writing – review & editing, Writing – original draft, Visualization, Methodology, Formal analysis, Data curation. **Chia-Chi Chen:** Visualization, Validation, Methodology, Investigation, Formal analysis, Data curation. **Li-Ya Lee:** Project administration, Methodology. **Shih-Wei Lin:** Resources, Project administration. **Yen-Lien Chen:** Supervision, Conceptualization. **Chin-Chu Chen:** Writing – review & editing, Supervision.

## Institutional review board statement

All experiments were fully approved from the Institutional Animal Care and Use Committee of the Agricultural Technology Research Institute. IACUC- No. 112058 for the colitis study, IACUC- No. 112019 for the constipation study, and IACUC- No. 110075 for gastric ulcer study.

## Data availability

The data and raw materials presented in this study are available on request from the corresponding author.

## Funding

This research did not receive any specific grant from funding agencies in the public.

## Declaration of competing interest

Grape King Bio Ltd. provided salaries and research materials to You-Shan Tsai, Li-Ya Lee, Shih-Wei Lin, Yen-Lien Chen, and Chin-Chu Chen. The company had no other involvement in the research process. The remaining authors declare no conflicts of interest.

## Data Availability

Data will be made available on request.
