# Supplementary material for: Health-promoting effects of Clostridium butyricum GKB7 on the gastrointestinal tract in murine models
Source: Biochem Biophys Rep. 2025 Jul 11;43:102145. doi: 10.1016/j.bbrep.2025.102145 (PMC12275026; doi:10.1016/j.bbrep.2025.102145)
Supplement: Multimedia component 1 [file mmc1.docx]

**Supplementary**

**Table S1. Stool water content on the first and the second week**

| Group | 1^st^ week  (Day 3 & 6) | 2^nd^ week  (Day 9 & 12) |
| --- | --- | --- |
| Normal | 64.44 ±0.45 | 63.64 ±0.55 |
| Loperamide | 63.79 ±0.38 | 63.51 ±0.79 |
| Loperamide +GKB7 | 62.98 ±1.03 | 64.61 ±0.95 |

Data are expressed as mean ±SEM.

**Table S2. Ulcer area in aspirin-induced stomach injury**

|  | Normal | Aspirin | Aspirin  + Omeprazole | Aspirin +GKB7 |
| --- | --- | --- | --- | --- |
| Ulcer area  (mm^3^) | 4.59 ±1.59 | 15.20 ±2.29 | 8.80 ±1.38 | 12.46 ±2.09 |

Data are expressed as mean ±SD.
